# The prevalence of ACPA is lower in rheumatoid arthritis patients with an older age of onset but the composition of the ACPA response appears identical

**DOI:** 10.1186/s13075-017-1324-y

**Published:** 2017-05-31

**Authors:** Debbie M. Boeters, Lukas Mangnus, Sofia Ajeganova, Elisabet Lindqvist, Björn Svensson, René E. M. Toes, Leendert A. Trouw, Tom W. J. Huizinga, Francis Berenbaum, Jacques Morel, Solbritt Rantapää-Dahlqvist, Annette H. M. van der Helm-van Mil

**Affiliations:** 1 Department of Rheumatology C1-R, Leiden University Medical Center, PO Box 9600, Leiden, 2300RC The Netherlands; 2 Department of Medicine Huddinge, Karolinska Institutet, Stockholm, Sweden; 3 Department of Clinical Sciences, Section of Rheumatology, Lund University and Skåne University Hospital, Lund, Sweden; 4 Department of Clinical Sciences, Section of Rheumatology, Lund University, Lund, Sweden; 5 Department of Rheumatology, Sorbonne University, INSERM UMR_S938, DHU i2B, Assistance Publique-Hôpitaux de Paris, Saint-Antoine Hospital, Paris, France; 6 Department of Rheumatology, Teaching Hospital Lapeyronie and Montpellier University, Montpellier, France; 7 Department of Public Health and Clinical Medicine/Rheumatology, University Hospital, Umeå, Sweden

**Keywords:** Rheumatoid arthritis, Age, Autoantibodies, ACPA characteristics

## Abstract

**Background:**

Rheumatoid arthritis (RA) consists of two syndromes, one autoantibody-positive and one autoantibody-negative. Existing data on the relation between age of onset and prevalence of autoantibodies were conflicting. Therefore this multicohort study assessed the age of onset in relation to the presence of autoantibodies. The association with characteristics of the anti-citrullinated protein antibodies (ACPA) response was also explored.

**Methods:**

The 1987 criteria-positive RA patients included in the Leiden EAC, BARFOT, ESPOIR, Umeå and Lund cohorts (*n* = 3321) were studied at presentation for age of onset and the presence of ACPA, rheumatoid factor (RF) and anti-carbamylated protein (anti-CarP) antibodies. Logistic regression analyses were performed; effect sizes were summarized in inverse-weighted meta-analyses. Within ACPA-positive RA, ACPA level was studied in all cohorts; ACPA isotypes, ACPA fine specificity and ACPA avidity index and clinical characteristics were studied in the Leiden EAC.

**Results:**

From the age of 50 onward, the proportion of ACPA-negative RA patients increased with age in the five cohorts. Similar observations were made for RF and anti-CarP. The composition of the ACPA response did not change with increasing age of onset with respect to titer, isotype distribution, fine specificity and avidity index. With increasing age of onset, RA patients smoked less often, had higher acute phase reactants and more often had a sub(acute) symptom onset.

**Conclusions:**

Data of five cohorts revealed that with older age of onset ACPA-negative RA is more frequent than ACPA-positive RA, while characteristics of ACPA-positive RA as judged by the composition of the ACPA response appeared not age dependent. Further biologic studies are needed to characterize the pathogenesis of ACPA-negative polyarthritis at older age and to promote personalized treatment decisions in ACPA-negative patients in daily practice.

**Electronic supplementary material:**

The online version of this article (doi:10.1186/s13075-017-1324-y) contains supplementary material, which is available to authorized users.

## Background

Rheumatoid arthritis (RA) is a syndrome for which characterization is based on a combination of clinical features. Symmetric polyarthritis of hands and feet is a key clinical feature and is captured in the 1987 classification criteria [1]. It is presumed that different biologic pathways can end up in the same clinical phenotype of RA. To arrive at personalized medicine, it is relevant to identify such different groups of patients. The most commonly used division is that into anti-citrullinated protein antibodies (ACPA)-positive RA and ACPA-negative RA, and both subgroups have differences in genetic and environmental risk factors [2, 3].

In addition to ACPA, there is some evidence suggesting that there are different characteristics of RA patients presenting at an older age. Several studies have shown that RA patients with disease onset at older age have a more equal gender distribution, more frequently an acute onset of symptoms [4, 5] and more often experience constitutional symptoms than younger patients at disease presentation [4–6]. ACPA positivity is more frequent with older age, suggesting that ACPA-positive RA may also be more frequent with older age [7]. However, within autoantibody-positive patients it was recently observed that patients with two or three autoantibodies were younger at onset than patients with one autoantibody [8]. In addition, some studies showed lower frequencies of autoantibodies in RA patients presenting at older age [9–14], while other studies observed no differences [15, 16] or showed a nonsignificant trend toward a higher prevalence of ACPA in RA patients presenting at older age [17, 18]. Altogether, the association between age of onset and the distribution of ACPA-positive RA versus ACPA-negative RA remains to be established.

If there is an association between age of onset and the presence of autoantibodies, this could be explained by different scenarios. There could be an age-related effect on the ACPA response itself. Then, in addition to the presence of ACPA, characteristics of the ACPA response could also vary with age. This could be a conceivable explanation because in the general population the immune system changes with ageing. For instance, an increase in proinflammatory cytokines, a more active innate immunity and a decline in the function of the adaptive immune system has been observed with older age [19]. T-cell senescence has been described and may mediate the development of RA [20]. With regard to B cells and antibodies, titers of antibodies against nearly all vaccines, including tetanus, decrease with age [21]. Furthermore there is a defect in isotype switching and limited production of high-affinity antibodies with increasing age, all thought to associate with decreased protection by vaccines and increased susceptibility to infections [22]. Whether B-cell senescence has a role in RA development is still unclear [19]. Despite these studies on the autoantibody response and aging, to our knowledge it is unknown whether age influences characteristics of the ACPA response, measured at RA onset.

An alternative explanation could be that some of the patients presenting at older age with ‘typical RA’ (e.g., symmetric polyarthritis of small joints) have differences in underlying biologic mechanisms compared with younger patients. Although biologic studies are needed to verify this hypothesis, detailed phenotypic studies can identify subtle differences between patient groups, despite their similarity in key clinical characteristics that are required for classification.

As a follow-up on previous studies of ACPA and age of onset as well as on the mentioned considerations, this study had three aims. Firstly, to determine the association between age of RA onset and the frequency of three autoantibodies (ACPA, rheumatoid factor (RF) and anti-carbamylated protein (anti-CarP) antibodies). For this purpose a large study on data of five cohorts was performed. Secondly, to study whether age at onset was associated with characteristics of the ACPA response in ACPA-positive RA patients. Thirdly, to substantiate previously reported associations between age at onset and clinical characteristics [4–6].

## Methods

### Patients

The association between age at RA onset and prevalence of ACPA and RF was studied in all five RA cohorts (Leiden Early Arthritis Clinic (EAC), BARFOT, ESPOIR, Umeå, Lund) and anti-CarP was studied in two cohorts (Leiden EAC, BARFOT). The association between age at RA onset and ACPA level was also studied in all five cohorts. Other ACPA characteristics were studied in ACPA-positive RA patients from the Leiden EAC. RA was defined as fulfilling the 1987 classification criteria [1]. The 2010 classification criteria were not used because autoantibodies are heavily weighted in these criteria, which may induce circularity between the parameter that was studied and the reference.

#### Leiden EAC

The Leiden EAC is an inception cohort set up in the Leiden University Medical Center (the Netherlands) that started in 1993 and includes patients with clinically confirmed arthritis and symptom duration < 2 years at presentation to the rheumatologist. [23] This department is the only referral center in a health care population of >400,000 inhabitants. At baseline questionnaires, joint counts and blood samples were collected. Information on smoking (present versus none and past) was obtained at baseline. The presence of shared epitope alleles was determined as described previously [24]. The patients studied were included between 1993 and 2015; a total of 1244 RA patients were consecutively included and studied here. The age ranged between 18 and 92 years.

#### BARFOT

The BARFOT (Better Anti-Rheumatic Farmaco-Therapy) study is an observational study of patients with early RA in Sweden [25]. Patients aged 18–93 years were included from six rheumatology centers when they were diagnosed with RA and had symptom duration < 1 year. In this study, 839 patients included between 1993 and 1999 were enrolled.

#### ESPOIR

The Evaluation et Suivi de POlyarthrites Indifférenciées Récentes (ESPOIR) is a cohort in which patients from 14 regional centers across France (16 university hospital rheumatology departments) were recruited [26]. Patients were aged 18–70 years and had ≥2 swollen joints for >6 weeks and <6 months and a high clinical suspicion on RA based on expert assessment. In total, 632 RA patients included between 2002 and 2005 were studied here.

#### Umeå

Umeå is an observational inception cohort in which 459 RA patients with symptom duration < 12 months from four different counties in Sweden were included between 1995 and 2010 [12]. The age ranged between 18 and 83 years.

#### Lund

This cohort study recruited patients from primary care units in the area of Lund, Sweden, and included patients with RA for <24 months aged 18–78 years [27]. Although at inclusion RA was defined using the 1958 criteria, these patients also fulfilled the 1987 criteria [28–30]. In total, 183 patients were included between 1985 and 1989; of these, 147 were previously evaluated in longitudinal studies [31, 32] and also studied here.

### Serological measurements

Baseline serum samples were tested for ACPA: Leiden EAC, anti-CCP2 Immunoscan RA Mark 2 (Eurodiagnostica, Arnhem), cutoff 25 U/ml, and anti-CCP2 EliA CCP (Phadia, Nieuwegein, the Netherlands), cutoff 7 U/ml, were used to determine the presence of ACPA, ACPA level studied in samples tested with the anti-CCP2-test from Eurodiagnostica; ESPOIR, anti-CCP2 (DiaSorin, France), cutoff 50 U/ml; BARFOT and Umeå, anti-CCP2 (Eurodiagnostica, Malmö, Sweden), cutoff 25 U/ml; and Lund, anti-CCP2 (Anamar Lund, using commercial kits, Inova Diagnostics, San Diego, CA), cutoff 20 U/ml. IgM RF was determined as follows: Leiden EAC, in-house ELISA; BARFOT, Serodia RA agglutination test (Fujirebio Inc., Tokyo, Japan); ESPOIR: Elisa, Menarini, France; Positive >9 UI/ml); Umeå, in-house ELISA; and Lund (ELISA, Anamar Lund, using commercial kits, Inova Diagnostics, San Diego, CA). IgG anti-CarP antibodies against carbamylated fetal calf serum were determined as described previously in the Leiden EAC [33], cutoff for positivity was based on the mean + 2SD from a set of 204 healthy controls; and in BARFOT the cutoff was based on the 82 controls from the source population [34].

### ACPA characteristics

Data on ACPA isotypes, ACPA fine specificity and ACPA avidity were determined as described previously [35] in 157 RA patients included in the Leiden EAC. In short, by measuring ACPA isotypes different antibody subclasses can be distinguished which all differ in their ability to mediate effector responses [38]. ACPA IgG1, IgG2, IgG3, IgG4, IgA and IgM were determined using a sandwich ELISA [37]. The total number of ACPA isotypes in each ACPA-positive patient was used in our analysis. ACPA fine specificity was studied to measure an increase or shift in antigen recognition. To determine ACPA fine specificity, antibodies against the citrullinated and the arginine-containing form of two peptides derived from vimentin (Vim1–16; Vim59–74), two peptides derived from fibrinogen (Fibα 27–43; Fibβ 36–52) and one peptide derived from α-enolase (Eno 5–20) and against citrullinated myelin basic protein were determined by in-house ELISA [36]. The sum of citrullinated antigens recognized by ACPA in each patient was used in our analysis. Finally the avidity of ACPA IgG, as a measure of the strength of the ACPA response, was determined with elution ELISAs [35]. Avidity is presented as the relative avidity index, which was defined as the ratio of the amount of residual antibodies bound to the antigen-coated plate after NaSCN (1 M) elution to the amount of bound antibodies in the absence of NaSCN, expressed as a percentage.

### Statistical analysis

**Fig. 1:**
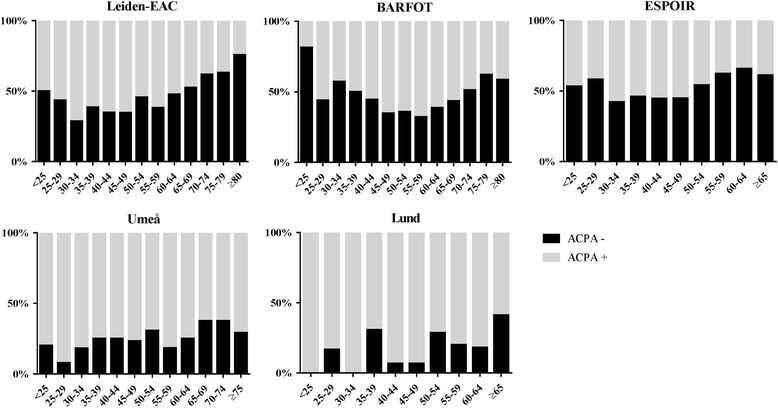
Proportion of ACPA-negative RA patients at different ages of RA onset. Proportion of ACPA-negative and ACPA-positive RA patients within the different age groups at RA onset in the five different cohorts. Number of patients in each age group: Leiden EAC: <25, *n* = 48; 25–29, *n* = 23; 30–34, *n* = 49; 35–39, *n* = 65; 40–44, *n* = 86; 45–49, *n* = 121; 50–54, *n* = 125; 55–59, *n* = 144; 60–64, *n* = 153; 65–69, *n* = 122; 70–74, *n* = 131; 75–79, *n* = 92; ≥80, *n* = 49; BARFOT: <25, *n* = 16; 25–29, *n* = 25; 30–34, *n* = 42; 35–39, *n* = 40; 40–44, *n* = 45; 45–49, *n* = 66; 50–54, *n* = 92; 55–59, *n* = 90; 60–64, *n* = 75; 65–69, *n* = 92; 70–74, *n* = 82; 75–79, *n* = 66; ≥80, *n* = 29; ESPOIR: <25, *n* = 30; 25–29, *n* = 24; 30–34, *n* = 45; 35–39, *n* = 52; 40–44, *n* = 65; 45–49, *n* = 78; 50–54, *n* = 107; 55–59, *n* = 109; 60–64, *n* = 73; ≥65, *n* = 49; Umeå: <25, *n* = 20; 25–29, *n* = 13; 30–34, *n* = 22; 35–39, *n* = 28; 40–44, *n* = 28; 45–49, *n* = 43; 50–54, *n* = 62; 55–59, *n* = 60; 60–64, *n* = 72; 65–69, *n* = 48; 70–74, *n* = 32; ≥75, *n* = 31; Lund: <25, *n* = 2; 25–29, *n* = 6; 30–34, *n* = 2; 35–39, *n* = 13; 40–44, *n* = 15; 45–49, *n* = 30; 50–54, *n* = 21; 55–59, *n* = 25; 60–64, *n* = 11; ≥65, *n* = 17. *ACPA* anti-citrullinated protein antibodies

To visually inspect the relation between age of onset and presence of autoantibodies, the proportion of autoantibody-positive and autoantibody-negative patients was plotted for different age groups of each 5 years. If <10 patients were present in the older age groups (BARFOT, Lund), age groups were summed. To statistically evaluate associations with age of onset, logistic regression analyses were performed per cohort with ACPA, RF or anti-CarP as the dependent variable and age of onset as the independent variable. Because descriptive results (Fig. 1, Additional file 1: Figure S1 and Additional file 2: Figure S2) showed that the proportion of autoantibody-positive RA patients decreased after ±50 years of age, a two-phase logistic regression analysis with one change point at 50 years was fitted. Odds ratios of the different cohorts (obtained from regression analyses) were entered in an inverse-weighted meta-analysis. This method weights results with a low standard error stronger than results with a higher standard error, thereby preventing over-representation of less precise data. A random effect model was used. The meta-analysis was performed separately for age of onset <50.0 years and >50.0 years and separately in males and females.

The proportion of ACPA-positive and ACPA-negative RA patients within the Leiden EAC was then compared with ACPA probabilities from the general Dutch population [7]. Within different age categories the risk of being ACPA-positive within the Leiden EAC was divided by the risk of being ACPA-positive within the general Dutch source population, revealing a risk ratio. The same was done for the risk of being ACPA-negative. Risk ratios of ACPA-positivity and ACPA-negativity were plotted for different age categories.

Data on ACPA characteristics were depicted visually for different age groups of each 10 years, and evaluated statistically with linear regression analysis (ACPA level, ACPA avidity) and ordinal regression analysis (ACPA isotypes, ACPA fine specificity), using Bonferroni correction for multiple testing.

Within the Leiden EAC, the association between age of onset and smoking, SE alleles and symptom onset was analyzed with logistic regression analysis, and with C-reactive protein (CRP), erythrocyte sedimentation rate (ESR) and swollen joint count (SJC) with Spearman’s correlation coefficient, using Bonferroni correction for multiple testing. Symptom onset was considered sub(acute) when there was prompt onset (e.g., <1 week), and not a gradual or intermittent onset. All regression analyses were adjusted for gender. Analyses were performed using SPSS version 23.0 (IBM).

## Results

### Patient characteristics

Baseline characteristics of all included patients are presented in Table 1. The majority of the included patients were female and the mean age of onset in the different cohorts ranged from 48.9 to 56.7 years. Symptom duration ranged from 18.3 to 43.3 weeks with the longest symptom duration observed in Lund. Within Leiden EAC, BARFOT and ESPOIR about 50% of the included patients were ACPA-positive, while in Umeå and Lund the percentage of ACPA-positive patients was 73.9% and 80.3%.

**Table 1:** Baseline characteristics of patients with rheumatoid arthritis included in the cohorts studied

Variable	Leiden EAC	BARFOT	ESPOIR	Umeå	Lund
Total number of patients	1244	839	632	459	147
Age, mean (SD)	56.6 (15.5)	56.7 (15.4)	48.9 (12.2)	53.9 (14.5)	50.7 (11.5)
Female, *n* (%)	827 (66.5)	538 (64.1)	484 (76.6)	321 (69.9)	98 (66.7)
Symptom duration^a^ (weeks), median (IQR)	18.3 (9–36)	26.1 (17–39)	21.4 (13–33)	28.0 (16–39)	43.3 (28–61)
Smoking at baseline, *n* (%)	308 (25.9)	227 (27.1)	137 (21.7)	107 (23.9)	39 (30.7)
ACPA+, *n* (%)	638 (52.8)	418 (55.0)	291 (46.0)	339 (73.9)	114 (80.3)
RF+, *n* (%)	715 (58.0)	453 (59.6)	344 (54.4)	362 (79.0)	115 (81.0)
Anti-CarP+, *n* (%)	474 (42.3)	280 (34.7)	NA	NA	NA
ESR (mm/h), median (IQR)	31 (16–50)	30 (15–50)	23 (12–41)	22 (12–39)	28 (13–50)
CRP (mg/L), median (IQR)	14 (6–35.5)	19 (7–47.5)	10 (3–26)	10 (8–25)	15 (0–45.5)
SJC, median (IQR)	5 (3–10)	10 (6–14)	7 (4–11)	6 (3–10)	6 (3–10)
TJC, median (IQR)	6 (2–11)	7 (3–12)	8 (4–14)	5 (2–10)	7 (4–11)

*n* number of patients, *SD* standard deviation, *IQR* interquartile range, *ACPA* anti-citrullinated protein antibodies, *RF* rheumatoid factor, *anti-CarP* antibodies *ESR* erythrocyte sedimentation rate, *CRP* C-reactive protein, *TJC* tender joint count based on 68 joints (Leiden EAC) or on 28 joints (BARFOT, ESPOIR, Umeå) or Ritchie index (Lund), *SJC* swollen joint count based on 66 joints (Leiden EAC) or on 28 joints (BARFOT, ESPOIR, Umeå) or on 50 joints (Lund), *NA* not available

^a^Time between symptom onset and inclusion in cohort

### ACPA prevalence decreased in RA patients with an older age at onset

**Fig. 2:**
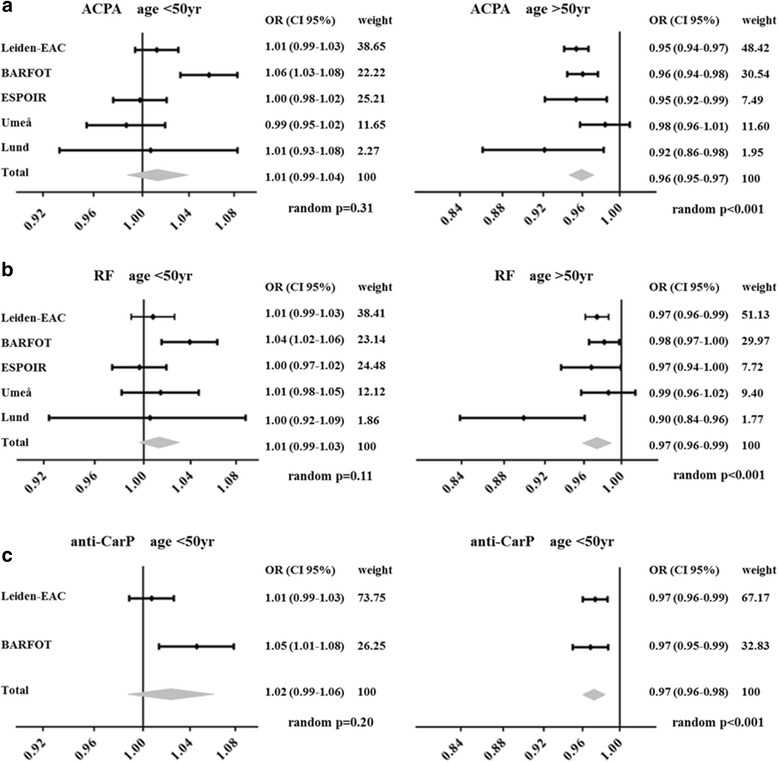
Meta-analysis on the association between age of onset (<50 and >50 years) and the presence of ACPA, RF and anti-CarP. Association between ACPA (**a**), RF (**b**) and anti-CarP (**c**) with age of onset in the different cohorts. The meta-analysis summarizes the effect of age of onset in the different cohorts and is based on a random effect model, combining ORs from separate logistic regression analyses of the different cohorts with age and gender as independent variables and ACPA, RF or anti-CarP as outcome. Separate meta-analyses were performed for the association between autoantibodies and age <50 years and >50 years. OR of 0.96 indicates that for a 1-year increase in age, the odds of being ACPA-positive decrease 4%; this is 18% per 5-year increase in age of RA onset (0.96^5^). *ACPA* anti-citrullinated protein antibodies, *anti-CarP* anti-carbamylated protein antibodies, *OR* odds ratio, *RF* rheumatoid factor

The proportion of ACPA-positive RA was plotted for all age categories in all five cohorts (Fig. 1). This showed that the proportion of ACPA-positive patients seemed to decrease after age of onset of 50 years. Logistic regression analyses with a change point at 50 years of age and with adjustment for gender were performed for each cohort; odds ratios (ORs) were combined in a meta-analysis. There was no association between the age of onset and the presence of ACPA in RA patients with an age of onset < 50 years (OR 1.01, 95% CI 0.99–1.04). However, age of onset > 50 years was associated with a lower frequency of ACPA-positivity (OR 0.96, 95% CI 0.95–0.97; Fig. 2a). An OR of 0.96 indicates that for a 1-year increase in the age of onset, the odds of being ACPA-positive decrease 4%; thus this reflects 18% per 5-year increase in age. Results were similar when studying ACPA in age categories of 5 years instead of continuously (Additional file 1: Figure S1). Similar results were observed for RF and anti-CarP (Additional file 2: Figure S2, Additional file 3: Figure S3, Fig. 2b, c).

Also when analyses were repeated with a change point at 60 years of age, similar results were obtained (meta-analysis: *p* < 0.001 for an association between ACPA presence and age of onset in patients aged > 60 years; and no significant association in patients aged < 60 years, *p* = 0.88).

**Fig. 3:**
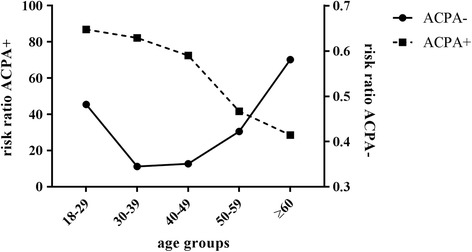
Risk of ACPA-positivity and ACPA-negativity in RA patients compared with individuals from the Dutch source population, presented for different age categories. For example, in the age group 18–29 the risk of being ACPA-positive was 87 times higher for RA patients than for individuals from the general Dutch population, and the risk of being ACPA-negative was 0.48 times higher (meaning 52% lower). The ratio for ACPA-negativity increased at older age. *ACPA* anti-citrullinated protein antibodies

Then we studied the proportion of ACPA-positive and ACPA-negative patients in relation to the ACPA prevalence of the Dutch source population (Fig. 3). This showed that, for example, in the age group 18–29 the risk of being ACPA-positive was 87 times higher for RA patients compared with individuals from the general population. In line with this, the risk of being ACPA-negative was 0.48 times higher (meaning 52% lower) for RA patients compared with individuals from the general population. The risk ratio for ACPA-negativity increased at older age.

### ACPA characteristics did not differ for different ages of RA onset

**Fig. 4:**
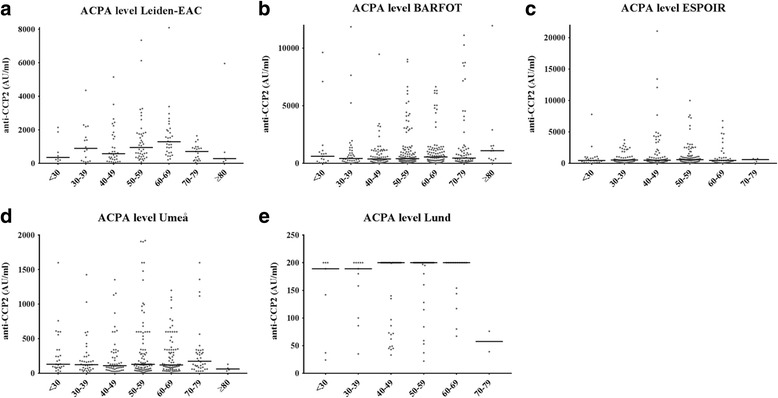
ACPA level of ACPA-positive RA at different ages of RA onset; data from five cohorts. Association between age of onset and ACPA level within RA patients of the Leiden EAC (**a**), BARFOT (**b**), ESPOIR (**c**), Umeå (**d**) and Lund (**e**) cohorts. In Lund the upper detection limit of the anti-CCP2 test was 200 U/ml; there were 76 patients with anti-CCP2 level > 200 U/ml. *Horizontal lines* represent median values. Each *dot* represents one patient. *ACPA* anti-citrullinated protein antibodies

**Fig. 5:**
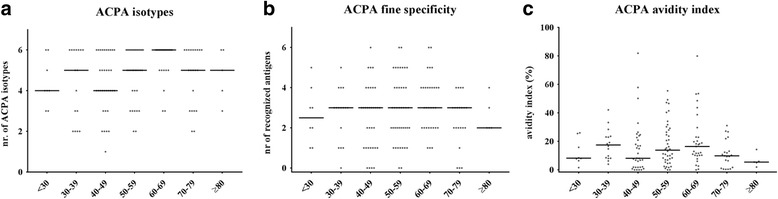
Isotypes, fine specificity and avidity index of ACPA-positive RA patients at different ages of onset; data from the Leiden EAC. Association between age of onset and ACPA isotypes (**a**), ACPA fine specificity (**b**) and ACPA avidity index (**c**) within RA patients of the Leiden EAC. *Horizontal lines* represent median values. Each *dot* represents one patient. *ACPA* anti-citrullinated protein antibodies

After having studied the presence of ACPA-positive RA, several characteristics of the ACPA response were evaluated within ACPA-positive RA patients. First, ACPA level was analyzed in relation to age of onset; no association between ACPA level and age of onset was observed (Leiden EAC *p* = 0.49, BARFOT *p* = 0.21, ESPOIR *p* = 0.91, Umeå *p* = 0.34, Lund *p* = 0.08; Fig. 4). Then within the Leiden EAC the number of ACPA isotypes was evaluated because isotype class switching can lead to an increased diversity of the antibody repertoire. The ordinal regression showed *p* = 0.03, and was not significant after correcting for multiple testing (cutoff Bonferroni correction *p* = 0.01, Fig. 5a). No association was observed between age at onset and the ACPA fine specificity (which we presented as the number of recognized citrullinated antigens by ACPA, *p* = 0.96; Fig. 5b) and the ACPA avidity index (which measures the overall binding strength of the ACPA response to CCP-2, *p* = 0.62; Fig. 5c). These findings together suggest that the analyzed ACPA characteristics were comparable within different age categories.

### Several clinical parameters in RA patients at disease onset associated with age of onset

The decrease in the relative proportion of ACPA-positive RA patients with increasing age of onset was not paralleled by age-related differences in the ACPA response itself, which suggests that an age-dependent effect on the ACPA response was not the most likely explanation. To further substantiate this, the associations of age with smoking and the HLA-SE alleles were determined, because these are the main risk factors for ACPA-positive RA. The presence of SE alleles remained constant over age of onset (*p* = 0.54), but the proportion of smokers decreased with increasing age (*p* < 0.001, Additional file 4: Figure S4). Similar to that observed for ACPA, this decrease was most prominent for RA patients with an age of onset > 50 years.

Another explanation for the higher proportion of ACPA-negative RA at older age of onset is that a group of (autoantibody-negative) patients with different etiopathology was preferentially present at older age and classified as RA. Because some previous studies have reported associations between clinical characteristics (male gender, more often acute onset, higher acute phase reactants) and an older age of onset [4–6], we aimed to substantiate this in the present data. We evaluated whether the association between age and the presence of autoantibodies was similar in males and females, showing that the effect was more pronounced in males (Additional file 5: Figure S5). Furthermore, an older age of onset was associated with higher CRP levels (ρ = 0.26, *p* < 0.001), higher ESR levels (ρ = 0.32, *p* < 0.001) and a higher number of swollen joints (ρ = 0.10, *p* = 0.001) at first presentation. RA patients presenting at older age also more often had (sub)acute onset of symptoms (*p* = 0.003, Additional file 6: Figure S6). These findings remained significant after Bonferroni correction (cutoff *p* = 0.008).

Altogether these data suggest that at older age there is a subgroup of patients who fulfill the classification criteria for RA that is more often male, nonsmoking, has higher acute phase reactants, more often has (sub)acute symptom onset and is also more often ACPA-negative.

## Discussion

Previous studies have evaluated differences in relation to the age of onset of RA, and have shown that some clinical characteristics were more prevalent at an older age of onset. Whether or not the ratio of ACPA-positive and ACPA-negative RA was also different was unresolved until now because different studies have yielded contrasting results. This prompted us to perform the present study in 3321 RA patients from five RA cohorts. The combination of the present data clearly showed that the proportion of autoantibody-positive patients (i.e., ACPA, RF and anti-CarP) was lower in RA patients who presented at older age. We also studied characteristics of the ACPA response, and within ACPA-positive RA patients characteristics of this response did not appear to differ at different ages of onset. Hence, our results suggest that the composition of the ACPA response is not different, but only the proportion of ACPA-positive RA is lower at older age of onset. In other words, the data revealed that ACPA-negative RA was more prevalent at older age.

Some findings within RA patients are different from findings obtained in the general population. In the general population, ageing is associated with lower antibody levels in response to vaccination [21]. In this study there was no association between ACPA level and age of onset. In addition, in the population autoantibodies (such as antinuclear antibodies, RF and ACPA) are increasingly prevalent at older age [7, 39–41], whereas within RA patents we observed a higher proportion of ACPA-negative disease at older age. This difference also resulted in the observation made in Fig. 3.

Interestingly, not only the proportion of ACPA-positive RA decreased with an older age at onset but also the proportion of RA patients who smoked at disease onset. This observation corresponds to the prevalence of present smokers in the general population, which decreases around 50 years of age [42]. Smoking is a known risk factor for ACPA-positive RA [43] and it is intriguing to speculate that a decrease in smoking patients (compared with nonsmokers) mediates the lower proportion of ACPA-positive RA at older age.

The 2010 classification criteria for RA could not be used to classify RA in the present study because of circularity between the dependent and independent variables. According to the 1987 criteria, RA is mainly classified based on clinical features, among which is symmetric polyarthritis of small joints. Our data suggest that patients fulfilling the 1987 criteria at older age more often had slight differences in other baseline characteristics, because they were more often males, had higher acute phase reactants and more often had (sub)acute onset of symptoms. Cluster analysis using only clinical characteristics, however, was insufficient to cluster patients on the individual level (data not shown). Nonetheless, based on the present data we presume that part of the ACPA-negative RA patients presenting at older age constitute a subgroup with slight differences in clinical presentation but with more pronounced differences in underlying pathogenic mechanisms. Biologic studies are now warranted to further evaluate this hypothesis and to identify a distinct subgroup within the seronegative patients.

A potential limitation is that the five cohorts were not completely comparable and that two cohorts contained an overall higher percentage of ACPA-positive patients than the other cohorts. Probably this can be explained by differences in health care systems or settings. When for instance the presence of ACPA (or other characteristics of more severe disease) is considered more relevant in the referral process or for inclusion in cohorts, this could explain the higher percentage of ACPA-positive patients in these cohorts. Nonetheless, here the percentage of ACPA-negative patients was also higher at older age of onset. The measurement of ACPA was not centralized, which may have led to different misclassification in different cohorts. Furthermore, anti-CarP was determined in only two of the five cohorts. We observed that RF and anti-CarP also decreased with increasing age of onset, although less distinctly than ACPA. The different autoantibodies often occur in the same patients; therefore another limitation is that we have not studied whether the decrease of RF and anti-CarP was independent of the age-related decrease of ACPA.

A final limitation is that studies on ACPA fine specificity, ACPA isotypes and ACPA avidity index were less powered than those on ACPA level. However, it is known that ACPA level is highly associated with ACPA fine specificity and the number of ACPA isotypes [44]. Because ACPA level was determined in all cohorts and there was no tendency toward differences in ACPA level in patients aged > 50 years at RA onset, this may suggest that ACPA fine specificity and ACPA isotypes would also remain stable with increasing age of RA onset. In some cohorts, patients aged > 80 appeared to have lower ACPA levels, although this age group contained very few patients.

## Conclusions

Characteristics of the ACPA response in ACPA-positive RA patients did not appear to be age dependent, while data of five cohorts revealed that with older age of onset ACPA-negative RA is more frequent than ACPA-positive RA. Further biologic studies are needed to characterize the pathogenesis of ACPA-negative polyarthritis at older age and to promote personalized treatment decisions in ACPA-negative patients in daily practice.

## Additional files

Additional file 1: Figure S1. Showing proportion of RF-negative RA patients at different ages of RA onset; data from five cohorts. Presented are the proportion RF-negative and RF-positive RA patients within the different age groups in the five different cohorts. Number of patients in each age group: Leiden EAC: <25, *n* = 49; 25–29, *n* = 23; 30–34, *n* = 49; 35–39, *n* = 66; 40–44, *n* = 88; 45–49, *n* = 125; 50–54, *n* = 127; 55–59, *n* = 147; 60–64, *n* = 156; 65–69, *n* = 128; 70–74, *n* = 130; 75–79, *n* = 93; ≥80, *n* = 51; BARFOT: <25, *n* = 16; 25–29, *n* = 25; 30–34, *n* = 43; 35–39, *n* = 40; 40–44, *n* = 45; 45–49, *n* = 66; 50–54, *n* = 92; 55–59, *n* = 90; 60–64, *n* = 75; 65–69, *n* = 92; 70–74, *n* = 81; 75–79, *n* = 66; ≥80, *n* = 29; ESPOIR: <25, *n* = 30; 25–29, *n* = 24; 30–34, *n* = 45; 35–39, *n* = 52; 40–44, *n* = 65; 45–49, *n* = 78; 50–54, *n* = 107; 55–59, *n* = 109; 60–64, *n* = 73; ≥65, *n* = 49; Umeå: <25, *n* = 20; 25–29, *n* = 13; 30–34, *n* = 21; 35–39, *n* = 28; 40–44, *n* = 28; 45–49, *n* = 43; 50–54, *n* = 62; 55–59, *n* = 60; 60–64, *n* = 72; 65–69, *n* = 48; 70–74, *n* = 32; ≥75, *n* = 31; Lund: <25, *n* = 2; 25–29, *n* = 6; 30–34, *n* = 2; 35–39, *n* = 13; 40–44, *n* = 15; 45–49, *n* = 30; 50–54, *n* = 21; 55–59, *n* = 25; 60–64, *n* = 11; ≥65, *n* = 17. (TIF 149 kb)

Additional file 2: Figure S2. Showing proportion of anti-CarP-negative RA patients at different ages of RA onset; data from two cohorts. Presented are the proportion of anti-CarP-negative and anti-CarP-positive RA patients within the different age groups in the Leiden EAC and BARFOT cohorts. Number of patients in each age group: Leiden EAC: <25, *n* = 43; 25–29, *n* = 23; 30–34, *n* = 44; 35–39, *n* = 62; 40–44, *n* = 79; 45–49, *n* = 111; 50–54, *n* = 112; 55–59, *n* = 135; 60–64, *n* = 144; 65–69, *n* = 114; 70–74, *n* = 120; 75–79, *n* = 87; ≥80, *n* = 47; BARFOT: <25, *n* = 18; 25–29, *n* = 27; 30–34, *n* = 37; 35–39, *n* = 41; 40–44, *n* = 43; 45–49, *n* = 62; 50–54, *n* = 96; 55–59, *n* = 88; 60–64, *n* = 80; 65–69, *n* = 103; 70–74, *n* = 88; 75–79, *n* = 87; ≥80, *n* = 38. (TIF 53 kb)

Additional file 3: Figure S3. Showing association between age and ACPA within the Leiden EAC with age in categories of 5 years. Logistic regression analyses were performed with the presence of ACPA as the outcome variable and gender and age as independent variables. Age was studied as a categorical variable (age groups of 5 years), with the age group 50–54 as the reference group. The ORs for ACPA positivity decreased linearly with increasing age groups. (TIF 566 kb)

Additional file 4: Figure S4. Showing proportion of present smokers and presence of SE alleles at different ages of onset of RA; data from the Leiden EAC. Presented are the proportion of currently smoking RA patients (*n* = 308) versus not smoking (none and past smoking) RA patients (*n* = 880) (**a**) and the proportion of patients carrying one or two SE alleles (*n* = 467) versus no SE alleles (*n* = 272) (**b**) within different age groups in the Leiden EAC. Number of patients in each group: smoking: <25, *n* = 47; 25–29, *n* = 22; 30–34, *n* = 48; 35–39, *n* = 66; 40–44, *n* = 85; 45–49, *n* = 119; 50–54, *n* = 125; 55–59, *n* = 141; 60–64, *n* = 153; 65–69, *n* = 121; 70–74, *n* = 127; 75–79, *n* = 87; ≥80, *n* = 47; SE alleles: <25, *n* = 28; 25–29, *n* = 12; 30–34, *n* = 29; 35–39, *n* = 40; 40–44, *n* = 59; 45–49, *n* = 74; 50–54, *n* = 81; 55–59, *n* = 80; 60–64, *n* = 91; 65–69, *n* = 73; 70–74, *n* = 75; 75–79, *n* = 61; ≥80, *n* = 36. (TIF 16283 kb)

Additional file 5: Figure S5. Showing meta-analysis on the association between age of onset and the presence of ACPA, RF and anti-CarP in male and female RA patients. Association between ACPA (**a**), RF (**b**) and anti-CarP (**c**) with age of onset in the different cohorts for males and females separately. The meta-analysis summarizes the effect of age of onset in the different cohorts and is based on a random effect model, combining the ORs from separate logistic regression analyses of the different cohorts with age as the independent variable and ACPA, RF or anti-CarP as outcome. Only the meta-analyses on the association between autoantibodies and age > 50 years at RA diagnosis are shown. OR of 0.94 indicates that for a 1-year increase in age of onset, the odds of being ACPA-positive decrease 6%; this is 27% per 5-year increase in age of onset (0.94^5^). (TIF 5577 kb)

Additional file 6: Figure S6. Showing association between age of onset and onset of symptoms within RA patients of the Leiden EAC. (**a**) Results of logistic regression analyses of age at RA onset in relation to the onset of symptoms. OR of 1.01 indicates that per 1-year increase in the age of onset, the odds of having (sub)acute onset increase 1%. This reflects 12% (1.01^10^) per 10-year increase in age of onset and 25% (1.01^20^) per 20-year increase in age of onset. (**b**) Proportion of RA patients with (sub)acute onset of symptoms in three age groups (*p* = 0.003). Number of patients per age group: <40, *n* = 181; 40–60, *n* = 466; >60, *n* = 537. (TIF 2476 kb)

## Abbreviations

ACPA: Anti-citrullinated protein antibodies
anti-CarP: Anti-carbamylated protein
BARFOT: Better Anti-Rheumatic Farmaco-Therapy
CRP: C-reactive protein
Eno: Enolase
ESPOIR: Evaluation et Suivi de POlyarthrites Indifférenciées Récentes
ESR: Erythrocyte sedimentation rate
Fib: Fibrinogen
HLA: Human leukocyte antigen
Leiden EAC: Leiden Early Arthritis Clinic
OR: Odds ratio
RA: Rheumatoid arthritis
RF: Rheumatoid factor
SD: Standard deviation
SE: Shared epitope
SJC: Swollen joint count
Vim: Vimentin
