# Supplementary figures and images for: The prevalence of ACPA is lower in rheumatoid arthritis patients with an older age of onset but the composition of the ACPA response appears identical
Source: Arthritis Res Ther. 2017 May 31;19:115. doi: 10.1186/s13075-017-1324-y (PMC5452396; doi:10.1186/s13075-017-1324-y)

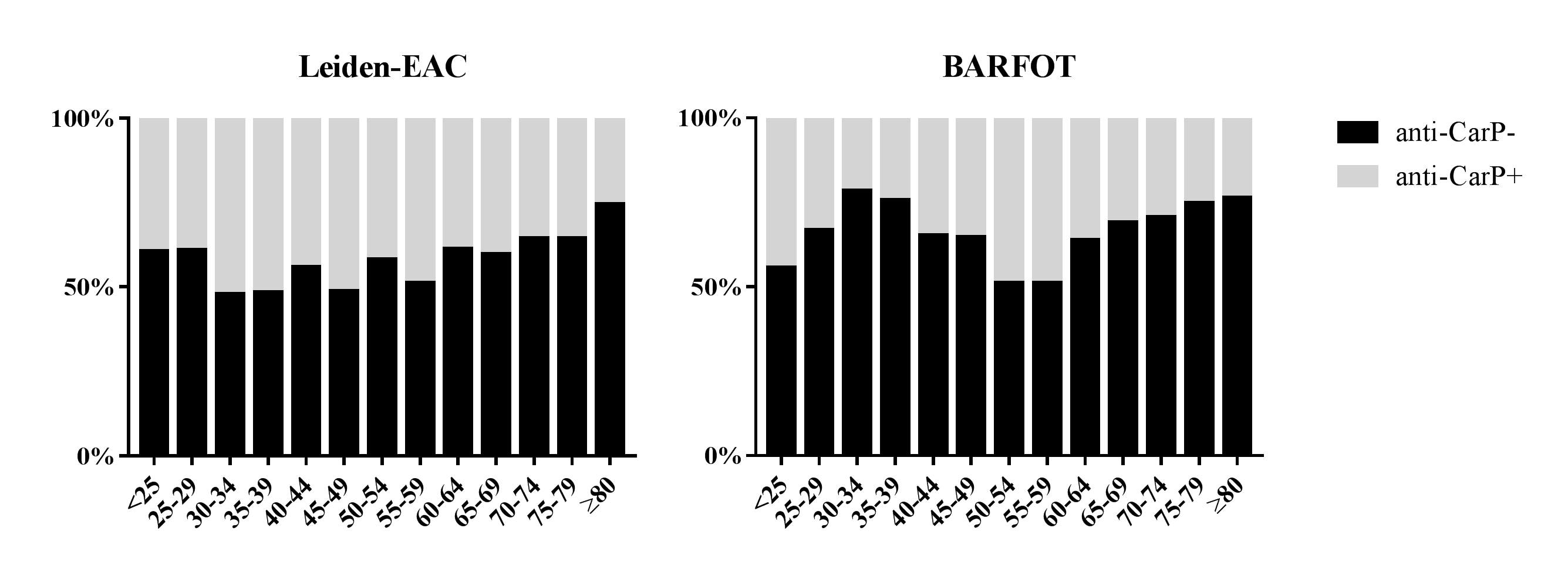

Supplement: Supplementary file 1 — Showing proportion of RF-negative RA patients at different ages of RA onset; data from five cohorts. Presented are the proportion RF-negative and RF-positive RA patients within the different age groups in the five different cohorts. Number of patients in each age group: Leiden EAC: <25, n = 49; 25–29, n = 23; 30–34, n = 49; 35–39, n = 66; 40–44, n = 88; 45–49, n = 125; 50–54, n = 127; 55–59, n = 147; 60–64, n = 156; 65–69, n = 128; 70–74, n = 130; 75–79, n = 93; ≥80, n = 51; BARFOT: <25, n = 16; 25–29, n = 25; 30–34, n = 43; 35–39, n = 40; 40–44, n = 45; 45–49, n = 66; 50–54, n = 92; 55–59, n = 90; 60–64, n = 75; 65–69, n = 92; 70–74, n = 81; 75–79, n = 66; ≥80, n = 29; ESPOIR: <25, n = 30; 25–29, n = 24; 30–34, n = 45; 35–39, n = 52; 40–44, n = 65; 45–49, n = 78; 50–54, n = 107; 55–59, n = 109; 60–64, n = 73; ≥65, n = 49; Umeå: <25, n = 20; 25–29, n = 13; 30–34, n = 21; 35–39, n = 28; 40–44, n = 28; 45–49, n = 43; 50–54, n = 62; 55–59, n = 60; 60–64, n = 72; 65–69, n = 48; 70–74, n = 32; ≥75, n = 31; Lund: <25, n = 2; 25–29, n = 6; 30–34, n = 2; 35–39, n = 13; 40–44, n = 15; 45–49, n = 30; 50–54, n = 21; 55–59, n = 25; 60–64, n = 11; ≥65, n = 17. (TIF 149 kb) [file 13075_2017_1324_MOESM1_ESM.tif]

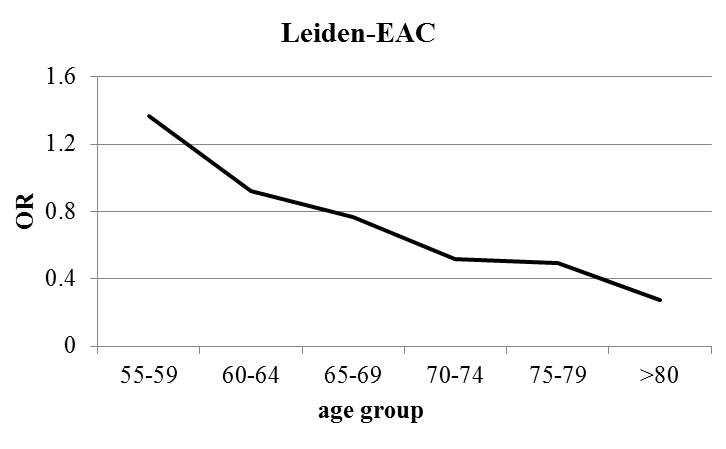

Supplement: Supplementary file 2 — Showing proportion of anti-CarP-negative RA patients at different ages of RA onset; data from two cohorts. Presented are the proportion of anti-CarP-negative and anti-CarP-positive RA patients within the different age groups in the Leiden EAC and BARFOT cohorts. Number of patients in each age group: Leiden EAC: <25, n = 43; 25–29, n = 23; 30–34, n = 44; 35–39, n = 62; 40–44, n = 79; 45–49, n = 111; 50–54, n = 112; 55–59, n = 135; 60–64, n = 144; 65–69, n = 114; 70–74, n = 120; 75–79, n = 87; ≥80, n = 47; BARFOT: <25, n = 18; 25–29, n = 27; 30–34, n = 37; 35–39, n = 41; 40–44, n = 43; 45–49, n = 62; 50–54, n = 96; 55–59, n = 88; 60–64, n = 80; 65–69, n = 103; 70–74, n = 88; 75–79, n = 87; ≥80, n = 38. (TIF 53 kb) [file 13075_2017_1324_MOESM2_ESM.tif]

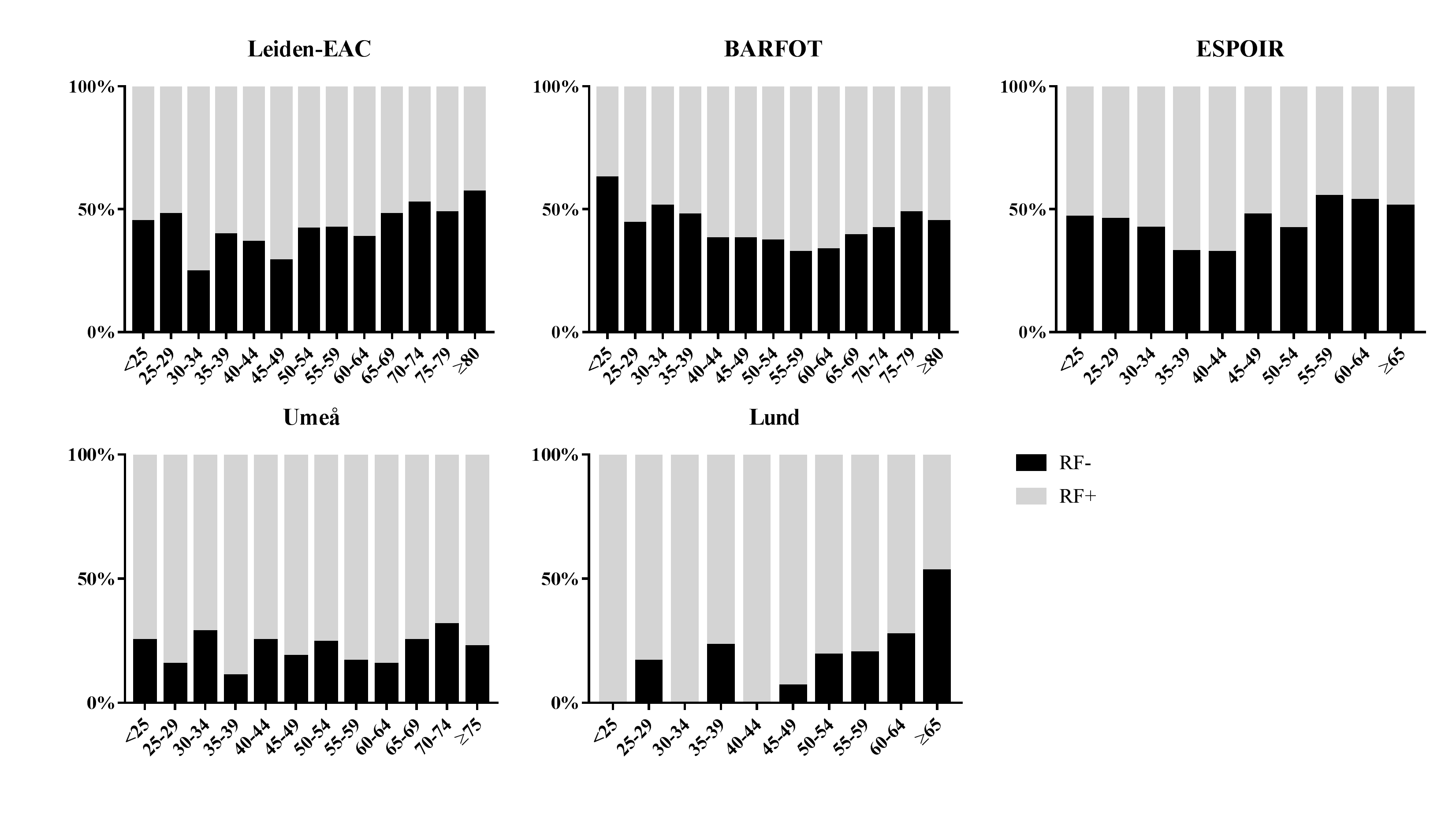

Supplement: Supplementary file 3 — Showing association between age and ACPA within the Leiden EAC with age in categories of 5 years. Logistic regression analyses were performed with the presence of ACPA as the outcome variable and gender and age as independent variables. Age was studied as a categorical variable (age groups of 5 years), with the age group 50–54 as the reference group. The ORs for ACPA positivity decreased linearly with increasing age groups. (TIF 566 kb) [file 13075_2017_1324_MOESM3_ESM.tif]

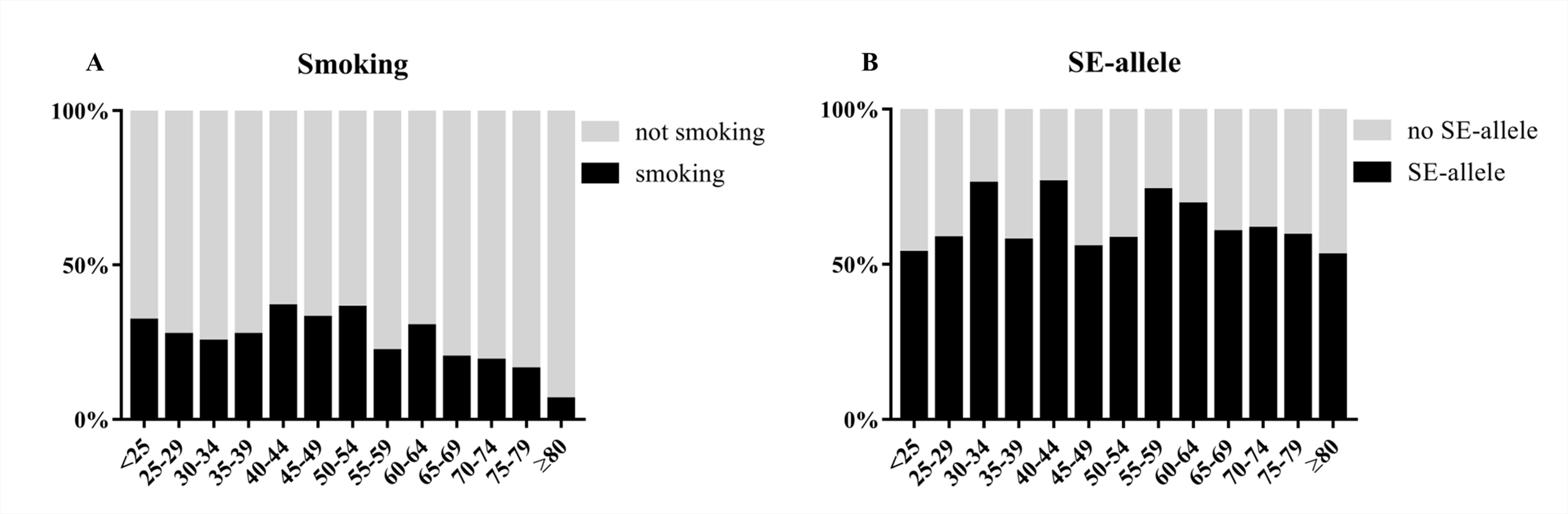

Supplement: Supplementary file 4 — Showing proportion of present smokers and presence of SE alleles at different ages of onset of RA; data from the Leiden EAC. Presented are the proportion of currently smoking RA patients (n = 308) versus not smoking (none and past smoking) RA patients (n = 880) (a) and the proportion of patients carrying one or two SE alleles (n = 467) versus no SE alleles (n = 272) (b) within different age groups in the Leiden EAC. Number of patients in each group: smoking: <25, n = 47; 25–29, n = 22; 30–34, n = 48; 35–39, n = 66; 40–44, n = 85; 45–49, n = 119; 50–54, n = 125; 55–59, n = 141; 60–64, n = 153; 65–69, n = 121; 70–74, n = 127; 75–79, n = 87; ≥80, n = 47; SE alleles: <25, n = 28; 25–29, n = 12; 30–34, n = 29; 35–39, n = 40; 40–44, n = 59; 45–49, n = 74; 50–54, n = 81; 55–59, n = 80; 60–64, n = 91; 65–69, n = 73; 70–74, n = 75; 75–79, n = 61; ≥80, n = 36. (TIF 16283 kb) [file 13075_2017_1324_MOESM4_ESM.tif]

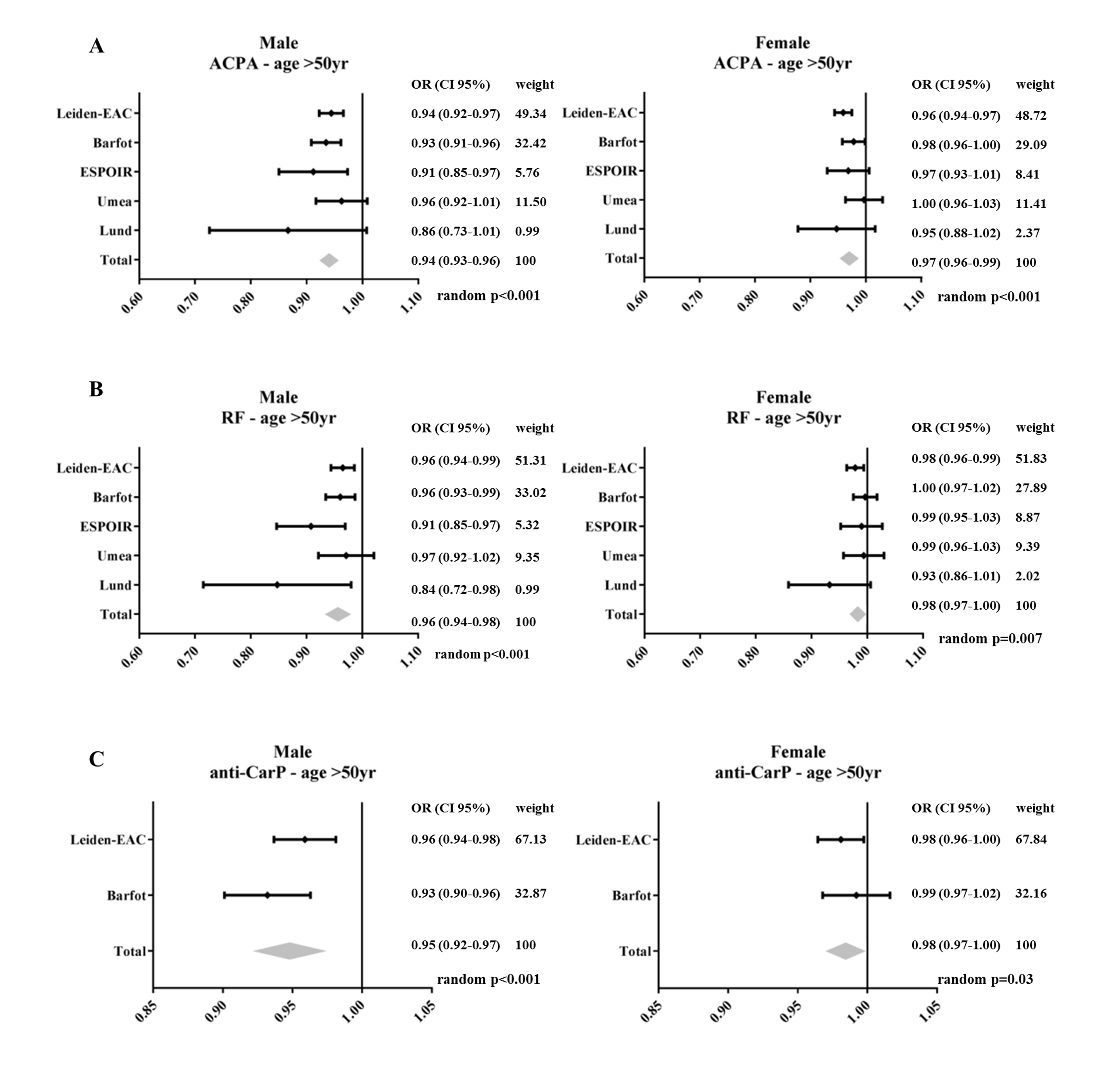

Supplement: Supplementary file 5 — Showing meta-analysis on the association between age of onset and the presence of ACPA, RF and anti-CarP in male and female RA patients. Association between ACPA (a), RF (b) and anti-CarP (c) with age of onset in the different cohorts for males and females separately. The meta-analysis summarizes the effect of age of onset in the different cohorts and is based on a random effect model, combining the ORs from separate logistic regression analyses of the different cohorts with age as the independent variable and ACPA, RF or anti-CarP as outcome. Only the meta-analyses on the association between autoantibodies and age > 50 years at RA diagnosis are shown. OR of 0.94 indicates that for a 1-year increase in age of onset, the odds of being ACPA-positive decrease 6%; this is 27% per 5-year increase in age of onset (0.945). (TIF 5577 kb) [file 13075_2017_1324_MOESM5_ESM.tif]

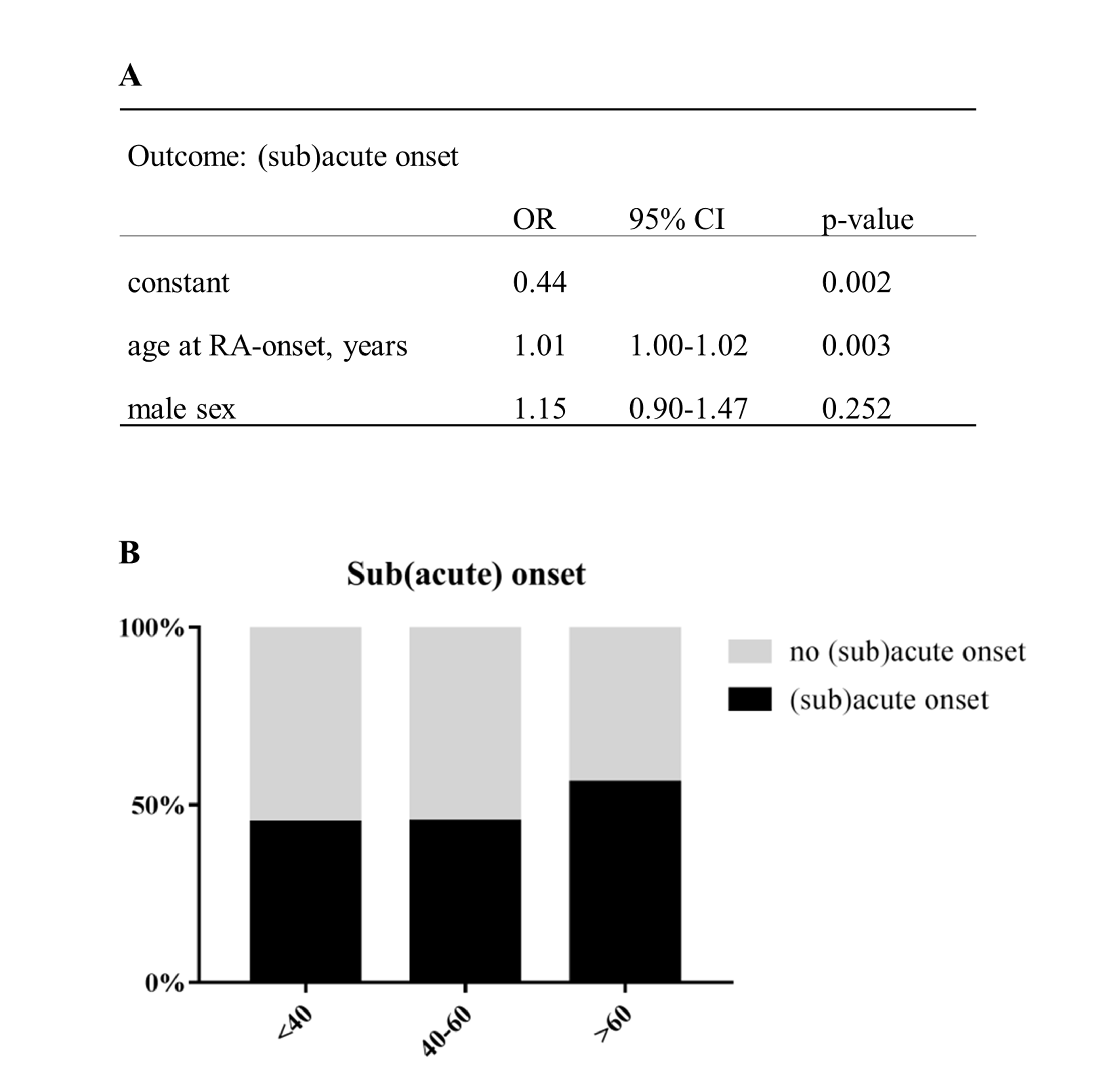

Supplement: Supplementary file 6 — Showing association between age of onset and onset of symptoms within RA patients of the Leiden EAC. (a) Results of logistic regression analyses of age at RA onset in relation to the onset of symptoms. OR of 1.01 indicates that per 1-year increase in the age of onset, the odds of having (sub)acute onset increase 1%. This reflects 12% (1.0110) per 10-year increase in age of onset and 25% (1.0120) per 20-year increase in age of onset. (b) Proportion of RA patients with (sub)acute onset of symptoms in three age groups (p = 0.003). Number of patients per age group: <40, n = 181; 40–60, n = 466; >60, n = 537. (TIF 2476 kb) [file 13075_2017_1324_MOESM6_ESM.tif]
